# Distinct patterns of mitochondrial genome diversity in bonobos (*Pan paniscus*) and humans

**DOI:** 10.1186/1471-2148-10-270

**Published:** 2010-09-02

**Authors:** Gábor Zsurka, Tatiana Kudina, Viktoriya Peeva, Kerstin Hallmann, Christian E Elger, Konstantin Khrapko, Wolfram S Kunz

**Affiliations:** 1 Division of Neurochemistry, Department of Epileptology and Life&Brain Center, University Bonn, Sigmund-Freud-Str. 25, 53105 Bonn, Germany; 2 Harvard Medical School, Beth Israel Deaconess Medical Center, 330 Brookline Avenue, Boston, MA 02215, USA

## Abstract

**Background:**

We have analyzed the complete mitochondrial genomes of 22 *Pan paniscus *(bonobo, pygmy chimpanzee) individuals to assess the detailed mitochondrial DNA (mtDNA) phylogeny of this close relative of *Homo sapiens*.

**Results:**

We identified three major clades among bonobos that separated approximately 540,000 years ago, as suggested by Bayesian analysis. Incidentally, we discovered that the current reference sequence for bonobo likely is a hybrid of the mitochondrial genomes of two distant individuals. When comparing spectra of polymorphic mtDNA sites in bonobos and humans, we observed two major differences: (i) Of all 31 bonobo mtDNA homoplasies, i.e. nucleotide changes that occurred independently on separate branches of the phylogenetic tree, 13 were not homoplasic in humans. This indicates that at least a part of the unstable sites of the mitochondrial genome is species-specific and difficult to be explained on the basis of a mutational hotspot concept. (ii) A comparison of the ratios of non-synonymous to synonymous changes (*d*_*N*_*/d*_*S*_) among polymorphic positions in bonobos and in 4902 *Homo sapiens *mitochondrial genomes revealed a remarkable difference in the strength of purifying selection in the mitochondrial genes of the F_0_F_1_-ATPase complex. While in bonobos this complex showed a similar low value as complexes I and IV, human haplogroups displayed 2.2 to 7.6 times increased *d*_*N*_*/d*_*S *_ratios when compared to bonobos.

**Conclusions:**

Some variants of mitochondrially encoded subunits of the ATPase complex in humans very likely decrease the efficiency of energy conversion leading to production of extra heat. Thus, we hypothesize that the species-specific release of evolutionary constraints for the mitochondrial genes of the proton-translocating ATPase is a consequence of altered heat homeostasis in modern humans.

## Background

The complete mitochondrial genome sequences of hominid species have been known for a while [1-3] and were frequently used in studies aiming to understand important aspects of human evolution [4,5]. While several thousand complete mitochondrial genomes from various modern human populations are available to date and complete mitochondrial genomes of six Neandertal individuals have been recently sequenced [4,5], the number of available individual mitochondrial sequences is very limited in non-human hominid species. The GenBank database contains at present only two complete mitochondrial genomes from *Gorilla gorilla *[GenBank:NC_011120, GenBank:NC_001645], two from *Pan troglodytes *[GenBank:NC_001643, GenBank:EU095335] and only a single mitochondrial genome from *Pan paniscus *[GenBank:NC_001644]. Studies addressing the mitochondrial DNA diversity of non-human hominids were, therefore, restricted to short regions of the mitochondrial genome, most typically parts of the non-coding and highly variable D-loop [6-9], but also cytochrome *c *oxidase subunit, COII [10], NADH dehydrogenase subunit, ND2 [11], or ribosomal genes [12]. These studies provided valuable information about the diversity of these species. However, analyses based on low numbers of polymorphic sites in short sequences are always at risk of being biased through high standard errors and gene-specific differences.

The power of analyzing a large number of complete mitochondrial genomes within a species was demonstrated in humans [13-15]. Analysis of within-species polymorphisms confirmed that purifying selection is the major force shaping human mitochondrial DNA diversity, but an impact of adaptive selection was suggested for haplogroups exposed to cold climates. It was hypothesized that a slightly decreased efficiency of mitochondria in synthesizing ATP by oxidative phosphorylation might be advantageous at cold temperatures due to dissipation of more heat [15]. Others questioned whether adaptive selection is the most plausible explanation for specific patterns of mtDNA diversity in human haplogroups [16]. A functional analysis of different mitochondrial genomes from African and Arctic haplogroups could not detect a potential bioenergetic relevance of the specific mtDNA variants [17].

Here we investigate the diversity of the mitochondrial genome in bonobos, and compare it to 4902 publicly available complete human mitochondrial genomes. Bonobos have been inhabiting a well defined territory in the Congo basin surrounded by rivers [9]. This means that, in contrast to *Homo sapiens*, the bonobo population did not undergo dramatic expansion and migration, and was not exposed to extreme climates. Therefore, the genetic diversity seen in this species can be largely attributed to random genetic drift within a rather stable population of individuals, and thus can serve as a good reference to unravel specific features of human evolution. Since our analysis included all 13 mitochondrial encoded subunits of oxidative phosphorylation complexes, we were able to investigate gene-specific differences of evolutionary constraints between *Pan paniscus *and *Homo sapiens*.

## Results

### Three clades of bonobo mtDNA phylogeny

To obtain a preliminary picture on the mtDNA diversity of the available bonobo samples, we sequenced the non-coding D-loop region and neighboring parts of the mitochondrial genome in a total number of 31 samples. Twenty-two individuals with distinct partial genotypes were then selected for complete mitochondrial genome sequencing. The sequences have been deposited in GenBank [GenBank:GU189657-GU189677, GenBank:HM015213]. Comparison of previously reported short D-loop sequences from 60 bonobo individuals [8,9] with the corresponding regions of the 22 new complete sequences demonstrates, as shown by a neighbor-joining tree (Additional file 1 Figure S1), that all major bonobo subgroups are represented in our sample. In order to be able to perform site-by-site comparisons with the 4902 *Homo sapiens *sequences that were available at the GenBank database at the date of this study, we aligned all sequences to the human mtDNA reference sequence (revised Cambridge Reference Sequence, rCRS [18]), and annotated polymorphic positions according to the rCRS numbering. We found 1287 differences to the rCRS that were stable in all bonobo sequences, and 364 positions that were polymorphic between bonobos.

**Figure 1:**
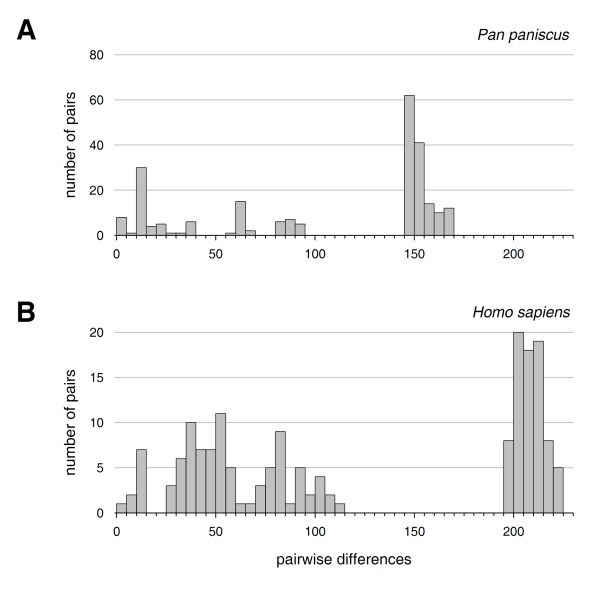
**Pairwise nucleotide differences of complete mitochondrial genomes**. (A) 22 *Pan paniscus *sequences. (B) *Homo sapiens *sequences including 21 selected contemporary sequences representing all major human haplogroups and 6 Neandertal sequences [4,5]. Rightmost bars indicate pairwise differences between modern humans and Neandertals.

Pairwise comparison of complete bonobo mitochondrial genomes (Figure 1A) showed a multimodal distribution, one set of pairs with less than 95 differences and a second, sharper peak representing 145 to 175 differences between sequences. This distribution is indicative of a stable demographic history, and suggests a lack of population expansion [9]. We compared the diversity of bonobo sequences with humans, by selecting 21 previously published sequences representing the major mitochondrial haplogroups. Additionally, recently published Neandertal mitochondrial genomes were included in the analysis (Figure 1B). Pairwise nucleotide differences show that the genetic diversity *within *the most diverse bonobo groups is comparable with the diversity of modern humans. The maximal nucleotide difference *between *bonobo groups is, however, 1.5 times higher than in humans, and thus somewhat closer to the distance between modern humans and the extinct Neandertal. Based on the molecular clock theory and assuming that the human-chimpanzee divergence happened 6 to 8 million years ago [19-21], Bayesian analysis of mtDNA sequences suggests that the most recent common ancestor (MRCA) of modern bonobos lived 540,000 (430,000- 660,000; 95% credibility interval) years ago.

**Figure 2:**
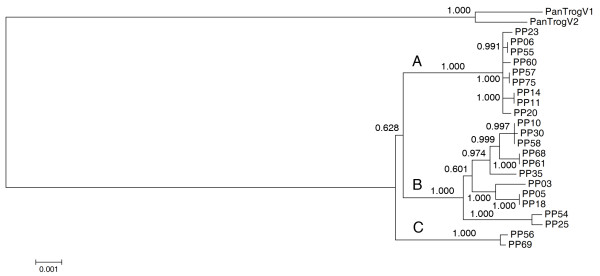
**Neighbor-joining tree of complete *Pan paniscus *mitochondrial genomes with *Pan troglodytes *as outgroup**. Bootstrap values are shown at the branches. Scale bar, evolutionary distance (substitutions per nucleotide position).

A more detailed picture of bonobo diversity is shown by the neighbor-joining tree of all 22 complete mitochondrial genomes using two *Pan troglodytes *genomes as outgroup (Figure 2). The detailed list of branch-specific mutation can be found in Additional file 2 Figure S2. Similarly to previous studies based on short D-loop sequences [8,9], most bonobo sequences belong to one of the two major groups ('A' and 'B' in Figure 2). Sequences belonging to haplogroup A show a low diversity between each other. It has to be mentioned, however, that two subgroups that are distinguishable within haplogroup A based on their HVRI (hypervariable region I) haplotypes [GenBank:AF176761, GenBank:AJ829459, GenBank:AJ829458, GenBank:AF137484, GenBank:AJ829461] and [GenBank:AF137486, GenBank:AF137485] (cf. Additional file 1 Figure S1) are not represented in our sample. The other large haplogroup ('B') displays a regular tree structure. Two D-loop sequences [GenBank:AF176762, GenBank:AF137491] (cf. Additional file 1 Figure S1) in previous studies showed a weak association with haplogroup B [9]. The corresponding two sequences (PP56 [GenBank:GU189665] and PP69 [GenBank: GU189670]) in our neighbor-joining tree of complete sequences were placed outside of the groups 'A' and 'B'. Since the two outlier sequences showed similar pairwise differences to sequences in haplogroups A and B, we conclude that PP56, PP69 and the two previously reported D-loop sequences [GenBank:AF176762, GenBank:AF137491] represent a third distinct linage ('C') of bonobos.

Before the present study, there was only a single complete bonobo mtDNA sequence available in public databases [GenBank:NC_001644] [1], which also served as reference sequence at early stages of our study. Neighbor-joining algorithms place this sequence within the haplogroup A when complete sequences are analyzed (data not shown). Indeed, the coding region of the database sequence (PPRefCOD in Additional file 2 Figure S2) carries all mutations that are present in one of our haplogroup A sequences (PP23). In contrast, the D-loop region of the database sequence (PPRefDL in Additional file 2 Figure S2) is clearly related to sequence PP10 that belongs to a subgroup within haplogroup B. Although, an extraordinary recombination event between the two major bonobo haplogroups could explain this discrepancy and cannot fully be excluded, we suspect that sequence [GenBank:NC_001644] is a hybrid sequence of two bonobo individuals. Therefore, we suggest it to be depreciated as reference sequence. Instead, the closest sequence, PP23 [GenBank:GU189661], could be used as the new reference sequence for the *Pan paniscus *mitochondrial genome.

### Species-specific homoplasies in bonobos

On the basis of the detailed mtDNA phylogeny of *Pan paniscus *we were able to identify 31 homoplasic sites (Table 1), i.e. nucleotide changes with multiple occurrences in independent bonobo lineages. According to current hypotheses, such homoplasies arise due to an increased instability of the specific positions ('hotspots' leading to recurrent or backward mutations [22]). We examined each of the bonobo homoplasies for the variability of the respective sites in humans. We found that among the 31 bonobo homoplasic positions 8 were stable in humans (either they were identical in all complete human mtDNA sequences, or occurred only in a single sequence or a single group). When classifying human polymorphic positions with very low allele frequency (< 0.25%) also as non-homoplasic, the amount of bonobo-specific homoplasies was even higher (13/31, 42%).

**Table 1:** Homoplasies in bonobos

	Bonobo homoplasy	Human frequency (N = 4902)	Stability in humans	Neighborhood difference (± 5 nt)
•	51C	1	private	
	151G	0	151C/T homoplasy	146C
	152C^a^	1347		
	153G	234		
	194T	115		
○	196C	10		
	198T	133		
	204C	309		
	ins309C^b^	1945		
•	341G	1	private	338T, 339T
	ins/del965C	49		961C
○	1809C	5		1811C
•	5573G	0	not present	
•	5910A	1	private	
•	6089G	0	not present	6092C
○	8227C	5		
	12127A	28		12124T, 12130C, 12131A
○	13488C	2		
○	14831A	10		14827T
•	16142T	6	single group	16138T
•	ins16170T	0	not present	
	16217C	192		16213A
	16249C	165		16244A
	16278T^a^	638		16273C
	16294T^a^	415		16289G, 16290A, 16292A
	16295T	56		16290A, 16292A
	16311C^a^	1030		
	16362C^a^	1231		
	16391A	41		
	16524G	26		16528T, 16529C
•	16559G	0	not present	

^a^Site classified as human homoplasy or ^b^hypervariable by Finnilä et al. [25]. Filled dots indicate bonobo-specific homoplasies (in case of positions marked by empty circles, the classification of human polymorphic positions as non-homoplasic was less conservative).

Some authors previously reported that species-specific mutational hotspots exist in primates [23], however, without being able to propose underlying molecular mechanisms. The only well recognized mechanism up to date to explain increased instability of certain sites in the mitochondrial genome is the neighborhood effect, when a nucleotide change affects the stability of some sites in its nearest vicinity [24]. To test whether the apparent species-specific instability of some of the positions in our investigated bonobo population might be caused by altered sequence context in the close vicinity of the particular sites, we searched for stable differences between humans and bonobos within a 5-nucleotide distance from homoplasic positions. Stable between-species nucleotide changes were detected in the vicinity of 5 bonobo-specific homoplasic sites (5/13, 38%), while the neighborhoods of the other 8 sites were identical between most humans and bonobos (Table 1). The ratio of neighborhood changes for the complete set of polymorphic bonobo sites was higher (210/364, 58%; difference not significant), clearly demonstrating that bonobo-specific homoplasic sites are not clustered around fixed inter-species nucleotide changes. Therefore, the neighborhood effect cannot explain the existence of more than half of the bonobo-specific homoplasies.

It is interesting to mention that most previously reported human homoplasic sites were apparently stable in our sample of bonobos. From the lists of human parallel mutations in the study of Finnilä et al. [25], none of the homoplasies in the coding region and only 5 out of 49 D-loop homoplasies were found to be homoplasic in bonobos. The human hypervariable length polymorphism at position 309 (alternatively numbered as 303) was also homoplasic in bonobos, but another hypervariable position, 16519, was identical in all bonobo individuals. None of the human coding region homoplasies reported by Elson et al. [26] was homoplasic in bonobos.

### Evolutionary constraints in hominid mitochondrial genomes

**Figure 3:**
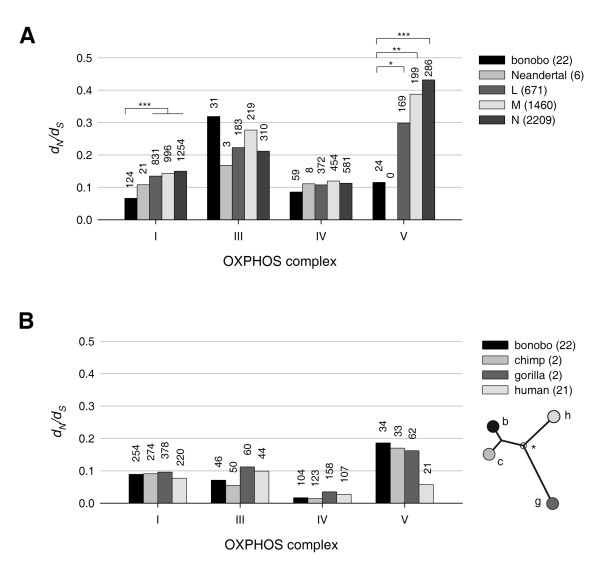
**Ratios of non-synonymous to synonymous mutations in mitochondrial protein coding genes**. The mitochondrial genes were pooled according to oxidative phosphorylation (OXPHOS) complexes: complex I (*MT-ND1, MT-ND2, MT-ND3, MT-ND4L, MT-ND4, MT-ND5, MT-ND6*), complex III (*MT-CYB*), complex IV (*MT-CO1, MT-CO2, MT-CO3*), and complex V (*MT-ATP8, MT-ATP6*). Numbers of individuals are shown in brackets; numbers above the bars indicate the total number of polymorphic position for the specific group of protein coding genes. (A) *d*_*N*_*/d*_*S *_ratios of within-group polymorphic sites in *Pan paniscus *and diverse human haplogroups, including Neandertals. Note that *d*_*N*_*/d*_*S *_ratio of within-species polymorphisms is also referred to as *θ_N_/θ_S _*in other studies. Statistical significance is indicated by stars (*, *P *< 0.05; **, *P *< 0.01; ***, *P *< 0.005) (B) *d*_*N*_*/d*_*S *_ratios of stable species-specific mutations in gorillas, chimpanzees, bonobos, and 21 human sequences representing all major haplogroups. The scheme on the left shows an unrooted tree of hominids. Filled circles represent species; nucleotide differences were calculated from the theoretical branching point indicated by star.

To investigate evolutionary constraints, we counted the number of within-species non-synonymous (*N*) and synonymous (*S*) mtDNA nucleotide changes in bonobos. We also determined these values for polymorphic mtDNA positions in recently published complete mtDNA sequences of Neandertals and publicly available mtDNA sequences of major human haplogroups (Figure 3A and Additional file 3 Figure S3). For each group, we calculated *d*_*N*_*/d*_*S *_values that describe the relation of the actual non-synonymous/synonymous ratio to the non-synonymous/synonymous ratio of all theoretically possible nucleotide changes in the specific sequence [27]. The mitochondrial encoded subunits of complexes III and IV showed comparable *d*_*N*_*/d*_*S *_ratios both in bonobos and the investigated human haplogroups. Values in complex III were found twice as high as in complex IV (*P *< 0.05 in bonobos; *P *< 0.0005 in humans), which is in line with previous reports from other organisms pointing to strong evolutionary constraints in cytochrome *c *oxidase [28]. The uniformly low *d*_*N*_*/d*_*S *_values for complex I in humans also suggest a strong purifying selection that is even more pronounced in bonobos. While the low *d*_*N*_*/d*_*S *_ratio of complex V genes in bonobos was comparable to those of complexes I and IV, the *d*_*N*_*/d*_*S *_ratio of complex V genes in humans showed notably elevated values (*P *< 5 × 10^-6^). Among the major human haplogroups, superhaplogroup L, containing most African clades, displayed the lowest *d*_*N*_*/d*_*S *_value (0.299), still 2.6-fold higher than in bonobos (*P *= 0.05). We observed the highest value in the superhaplogroup N, where most European haplogroups belong, with 0.432 (3.8-fold of that in bonobos; *P *= 0.0048). When examining smaller haplogroups in detail (Additional file 3 Figure S3), we found the lowest value in the group of San (haplogroup L0a'), the most ancient human haplogroup (0.249; 2.2-fold of that in bobonos; difference not significant), while the highest *d*_*N*_*/d*_*S *_value was observed in haplogroup A (0.879; 7.6-fold of that in bonobos; *P *= 0.0003). Haplogroup A is one of the major haplogroups that gave rise to Native American populations by migrating from Eastern Asia through the Bering land bridge [29,30]. Other haplogroups with similar demographic history (C and D) did not show such an exceptionally high *d*_*N*_*/d*_*S *_value (Additional file 3 Figure S3). No *d*_*N*_*/d*_*S *_value could be calculated for complex V in Neandertals, because none of the reported complete Neandertal sequences contained any mutation in the two mitochondrial genes for the ATPase subunits.

Next, we compared between-species stable nucleotide changes in gorillas, chimpanzees, bonobos, and humans (Figure 3B). This analysis confirmed the relevance of strong purifying selection against non-synonymous mutations in complex IV, between-species differences showing even lower *d*_*N*_*/d*_*S *_ratios than within-species polymorphisms. A similar decreased ratio of non-synonymous nucleotide changes at the between-species scale was observed for complex III, although, the evolutionary constraints were in general weaker compared to complex IV. Complex V contained the highest portion of between-species non-synonymous changes in bonobos, chimps and gorillas (significantly different from complex IV, *P *< 0.0005), but this value was much lower in humans (no significant difference from other complexes). This suggests that complex V was under a relatively strong constraint after the divergence of the human branch from the *Homo*/*Pan*/*Gorilla *common ancestor, but the strength of the purifying selection decreased dramatically after modern humans emerged and spread around the world.

## Discussion

In this study we present 22 complete mitochondrial genomes from individuals of the species *Pan paniscus*, the closest extant relatives of today's modern humans beside chimpanzees. Previous studies have explored the phylogeny of bonobos by investigating short sequences in many individuals [6-12], however, only comparison of complete mitochondrial genomes can provide means to reveal gene-specific differences in the degree of diversity and the strength of evolutionary constraints.

The phylogenetic tree of bonobos presented here, based on complete mitochondrial genomes, is in line with the previously published D-loop tree [8,9], demonstrating that HVRI sequencing can be a useful tool for investigating genetic diversity at small time scales. In accordance with the high variability of the D-loop [22], the majority of homoplasic sites, i.e. nucleotide changes that occur multiple times in independent bonobo lineages, were detected in the D-loop. Most of these homoplasic D-loop polymorphisms were also homoplasic in humans, thus, these sites can be considered as species-independent mutational hotspots. In contrast, many of the bonobo homoplasic positions in the much less variable coding region were stable in humans. Similarly, previously reported human coding region homoplasies were all found stable in bonobos. It is still not clear what causes certain sites to show apparent instability in one species while being stable in a closely related species. We investigated one possible mechanism for species-specific homoplasies, the effect of nucleotide changes in the close vicinity of polymorphic sites. We found that the majority of the bonobo-specific homoplasies were located in regions that were identical between humans and bonobos. Thus, a considerable portion of bonobo-specific homoplasies cannot be explained by between-species differences in the sequence context. Whether the broader mtDNA context or changes in the nuclear background can account for species-specific instability of certain mtDNA sites, or the apparent mtDNA instability might be a consequence of heteroplasmy-related phenomena [31,32], remains to be elucidated.

The comparison between the D-loop regions of the 22 new complete bonobo mitochondrial genomes, and a previously reported bonobo D-loop tree [8,9] revealed that, despite the limited number of individuals, the group of bonobos investigated in this study well represents the entire known bonobo population. Beside the two previously described haplogroups of bonobos [9], the analysis of complete mtDNA sequences made it possible to distinguish a third major bonobo lineage. Notably, this particular bonobo clade carried the lineage-specific mutation, m.8344A>G, in the mitochondrial gene for tRNA-Lys (*MT-TK*). This mutation is one of the most frequent pathogenic mtDNA mutations in humans, and associated with the MERRF syndrome (myoclonus epilepsy with ragged red fibers; MIM 545000 [33]). Apparently, the pathogenic effect of this particular mutation might be compensated by a neighboring mutation in the tRNA-Lys gene, m.8343A>G, that is present in all *Pan *sequences.

The number of pairwise nucleotide differences between members of the three major bonobo haplogroups was 1.5 times higher than the maximum pairwise differences between modern humans, placing the time of the most recent common ancestor of today's bonobos somewhat between that of modern humans and the Neandertals. This substantial diversity of mitochondrial genomes suggests that a recent population bottleneck hypothesized for humans [1], did not take place in bonobos. An important incidental finding, revealed by the phylogenetic tree, is that the only complete bonobo mtDNA sequence available to date in the GenBank database, also used as the bonobo mtDNA reference sequence, is probably an artificial hybrid of two sequences belonging to two different major haplogroups.

The sequence information on the entire mitochondrial genomes of 22 bonobo individuals allowed us to compare the genetic diversity of different mitochondrial genes and estimate possible evolutionary constraints acting on these genes. For this we grouped protein coding genes of the complexes of the oxidative phosphorylation (OXPHOS), and analyzed them for the ratio of within-species non-synonymous and synonymous nucleotide polymorphisms, a well established marker of evolutionary constraints. Any value of *d*_*N*_*/d*_*S *_below 1 indicates that the ratio of non-synonymous to synonymous changes is less than the ratio of theoretically possible non-synonymous to synonymous changes, and is considered to be a sign of negative selection against non-synonymous, potentially deleterious mutations [27]. Not only in bonobos but also in all analyzed human haplogroups, all four mitochondrial encoded OXPHOS complexes displayed values below 1, indicating that negative selection is the major force shaping the mtDNA diversity in these species.

When searching for species-specific changes in selection patterns, we found that genes coding for subunits of the proton-translocating ATPase (complex V of the oxidative phosphorylation) showed the most striking difference between bonobos and humans. While in bonobos the strength of within-species evolutionary constraints in the ATPase genes was comparable to that of complexes I and IV, most human haplogroups displayed *d*_*N*_*/d*_*S *_values in the ATPase genes that were higher than any of the other mitochondrial OXPHOS complexes. In contrast, between-species nucleotide changes showed high proportion of non-synonymous changes for ATPase in the branches of bonobos, chimps and gorillas, but were less frequent in humans (cf. last clusters of Figure 3A and 3B). We interpret these findings as evidence for a strong species-specific evolutionary constraint on the mitochondrial subunits of ATPase after the divergence of humans from the common *Homo/Pan/Gorilla *ancestor, which constraint was, however, released when modern humans migrated from Africa and populated the world.

This finding is further underlined by the potential severity of amino acid changes caused by the detected non-synonymous mutations in complex V. As shown in Additional file 4 Table S1, human haplogroup A displays a higher frequency of more severe amino acid changes than the most ancient human haplogroup (haplogroup L0a') or bonobos. Since complex V synthesizes ATP driven by proton translocation across the inner mitochondrial membrane, it is very likely that severe amino acid changes in both subunits of this complex might have a crucial influence on the efficiency of the energy conversion [34]. Lower efficiency would mean that a higher proportion of the electrochemical proton gradient is dissipated in the form of heat. In line with this, it was previously hypothesized that a mildly increased heat production by mitochondria might have an advantageous effect in adaptation to cold climates, i.e. ATPase polymorphisms could undergo positive selection [15].

Bonobos have been inhabiting a well defined territory in the Congo basin with rather constant climate conditions and with large rivers as natural borders [9]. Therefore, the effect of climate adaptation must be negligible in bonobos, in sharp contrast to humans, who successfully populated extreme climate zones. In light of our data suggesting released evolutionary constraints for the OXPHOS complex V, one might hypothesize that while spreading out of Africa, humans escaped an important environmental factor that was limiting their evolution. This factor could be high temperature (i.e. less efficient complex V and the resulting increased heat production could result in body overheating at high temperatures). Climate is, however, unlikely the only reason for the weaker purifying selection for complex V genes in humans. The human superhaplogroup L that includes most African lineages showed a more than twice as high *d*_*N*_*/d*_*S *_value as bonobos, despite the fact that these lineages, not leaving Africa, were also not exposed to extreme climates. Certain physiological changes (e.g. loss of body hair, high density of eccrine sweat glands, bipedalism) might have rendered increased heat production, caused by slightly deleterious ATPase mutation, less harmful in humans. This assumption is in line with hypotheses suggesting that improved heat homeostasis was an important factor in human evolution [35,36]. A further reason for the relaxation of purifying selection in humans might be the less intense sperm competition as compared to bonobos [37,38]. Although, a selection based on the energetic performance of the sperm is not expected to influence the transmission of mtDNA variants directly (because of the maternal inheritance of mtDNA), it still might have an indirect effect through the selection pressure on nuclear genes relevant for mitochondrial function.

Beside the difference of purifying selection in humans and bonobos, we also observed differences between human haplogroups. Of all investigated human clades, African haplogroups, and the San in particular, showed the lowest *d*_*N*_*/d*_*S *_values in the ATPase genes. The higher *d*_*N*_*/d*_*S *_values in other populations that left Africa could be accounted for environmental factors. Notably, the highest ATPase *d*_*N*_*/d*_*S *_value was observed in haplogroup A, one of the three major clades giving rise to the Native American population after migrating from Asia through the Bering land bridge. The *d*_*N*_*/d*_*S *_value in haplogroup A was close to 1, suggesting an almost complete lack of purifying selection in the ATPase genes. However, other haplogroups (C and D) that migrated through the same route did not show comparably high *d*_*N*_*/d*_*S *_values (Additional file 3 Figure S3). Therefore, we conclude, in agreement with others [16], that the observed increase of *d*_*N*_*/d*_*S *_values for the mitochondrial ATPase genes in humans cannot be interpreted in favor of positive selection at colder climate conditions, but rather is the result of the release of strong evolutionary constraints during population expansion and migration of modern humans.

## Conclusions

We have sequenced the complete mitochondrial genome of 22 *Pan paniscus *individuals. The analysis of the 22 new complete mtDNA sequences revealed the existence of three major lineages in the bonobo phylogeny. Out of these three lineages only two were identified previously, when the analysis was restricted to the highly polymorphic D-loop region of the mitochondrial genome. Comparing the new sequences with the only complete bonobo mtDNA sequence that had been available in public databases before the present study, we found that the database sequence is very likely a hybrid of sequences from two different bobono haplogroups. We analyzed the spectrum of nucleotide changes with multiple occurrences in independent bonobo lineages (homoplasies) and examined the stability of these homoplasic sites in humans. We found that 13 of 31 bonobo homoplasies were species-specific, and as such are difficult to be explained by the mutational hotspot hypothesis. The most striking difference between bonobo and human mtDNA diversity we observed when comparing the ratios of non-synonymous to synonymous mutations in different protein coding genes of the mitochondrial genome. The mitochondrial subunits of the proton-translocating ATPase complex showed a significantly increased rate of amino acid changing mutations within human haplogroups in comparison to bonobos. We hypothesize that changes of the ATPase complex that reduce the efficiency of ATP synthesis and increase heat production were rendered less harmful due to alterations of the heat homeostasis in humans.

## Methods

### DNA samples

Multiple buccal swab samples from bonobos living in zoos of Cologne and Frankfurt were obtained by keepers of the animals. Total DNA was isolated from buccal swabs using the QiaAmp DNA Mini Kit (Qiagen, Hilden, Germany). Further bonobo DNA samples were kindly provided by the EUPRIM-NET Project (Biomedical Primate Research Centre, Rijswijk, The Netherlands), and the Gene Bank of Primates (German Primate Centre, Göttingen, Germany). Additional samples were obtained by KK from Coriell Cell Repositories (Camden, New Jersey, USA). This study was approved by the Ethics Committee of the University Bonn (reference number 128/09).

### mtDNA sequencing

A set of 28 primer pairs was used to amplify overlapping mtDNA fragments from bonobo DNA samples. To avoid sample mix up, each individual was processed separately. Direct sequencing of the purified PCR products was carried out on an automatic sequence analyzer by a commercial sequencing service (MWG Biotech, Ebersberg, Germany). Sequence reads were confirmed manually and complete mtDNA sequences were assembled using the SeqMan module of the Lasergene 8.0 software (DNASTAR, Madison, WI, USA). Complete mtDNA sequences have been deposited in GenBank [GenBank:GU189657-GU189677 and GenBank:HM015213].

### Phylogenetic analyses

To be able to compare hominid mtDNA sequences to each other, as well as, to 4902 human database sequences, all sequences were aligned to the human reference sequence (revised Cambridge Reference Sequence, rCRS, NC_012920 [18]) with the ClustalX 2 software [39]. Alignments were verified and corrected manually using the MEGA 4.0 program [40]. The annotation of polymorphic positions, including nucleotide positions within genes and amino acid positions with eventual amino acid changes for protein coding genes, were done automated based on the rCRS annotation list using the core algorithm of the MitoWheel web application [41]. In cases of double annotations in the short overlapping regions between the genes *MT-ND4L *and *MT-ND4 *and between genes *MT-ATP8 *and *MT-ATP6*, polymorphisms were ranked according to the severer change. Total numbers of synonymous and non-synonymous sites were calculated using the DnaSP v4.50.3 program [42]. For the comparison of human haplogroups, sequences were grouped based on the presence or absence of group-defining mtDNA mutations. Haplogroup defining mutations are listed in Additional file 5 Table S2. Statistical significance was assessed by two-tailed Fisher's exact test. Neighbor-joining trees were generated with the ClustalX 2 program [39]. We estimated the posterior distribution of divergence times using the mcmctree program [43] with parameters as described for the analysis of the Neandertal mtDNA [4], assuming that the chimpanzee-human divergence happened 6-8 million years ago [19-21]. The analysis included, apart from the 22 new bonobo mtDNA sequences, two gorilla sequences, two chimpanzee sequences, and 21 human sequences representing all the major human haplogroups.

## Authors' contributions

GZ participated in the design of the study, performed the sequence and phylogenetic analyses, created the figures and wrote the paper. TK, VP and KH performed sequencing, analyzed the sequences and participated in drafting the manuscript. CEE and KK participated in the design of the study, collection of samples and revising the manuscript. WSK conceived of and designed the study, organized the collection of the samples, analyzed sequences and wrote the paper. All authors read and approved the final manuscript.

## Supplementary Material

Additional file 1 **Figure S1**. Neighbor-joining tree of *Pan paniscus *hypervariable region I sequences using *Pan troglodytes *as outgroup. Previously published sequences are shown by their GenBank accession numbers. Complete *Pan paniscus *mtDNA sequences described in this study are marked by dots. Scale bar, evolutionary distance (substitutions per nucleotide position). Click here for file

Additional file 2 **Figure S2**. Detailed phylogenetic tree of complete *Pan paniscus *mtDNA sequences displaying all detected polymorphic positions. For each branch, strictly branch-specific mutations are listed on the left-hand side of the line, while homoplasic mutations (occurring also in other independent branches) on the right-hand side. Stars indicate the lack of the specific bonobo-allele (representing a 'backwards' mutation to the human reference). A list of such 'backwards' mutating sites is shown at the root (top). PPRefCOD and PPRefDL, coding and D-loop regions of the only previously reported *Pan paniscus *mtDNA sequence [GenBank:NC_001644]. Click here for file

Additional file 3 **Figure S3**. Ratios of non-synonymous to synonymous mutations in mitochondrial protein coding genes. *d*_*N*_*/d*_*S *_ratios of within-group polymorphic sites in the mitochondrial encoded subunits of complexes I, III, IV and V in *Pan paniscus *and diverse human haplogroups. Numbers of analyzed individual sequences are shown in brackets. Click here for file

Additional file 4 **Table S1**. Non-synonymous mutations in mitochondrial genes of complex V. Scoring of amino acid changes according to Betts and Russell [44]. Positive values indicate favored changes, zero neutral changes, negative values disfavored changes in membrane proteins. Click here for file

Additional file 5 **Table S2**. Human haplogroup definitions used in the study. Haplogroups defined by the presence (+) or the absence (-) of specific mutations as compared to the revised Cambridge Reference Sequence [18]. Click here for file
