# Supplementary material for: Distinct patterns of mitochondrial genome diversity in bonobos (Pan paniscus) and humans
Source: BMC Evol Biol. 2010 Sep 2;10:270. doi: 10.1186/1471-2148-10-270 (PMC2942848; doi:10.1186/1471-2148-10-270)

Additional file 1 Figure S1 - Neighbor-joining tree of *Pan paniscus* hypervariable region I sequences using *Pan troglodytes* as outgroup. Previously published sequences are shown by their GenBank accession numbers. Complete *Pan paniscus* mtDNA sequences described in this study are marked by dots. Scale bar, evolutionary distance (substitutions per nucleotide position).

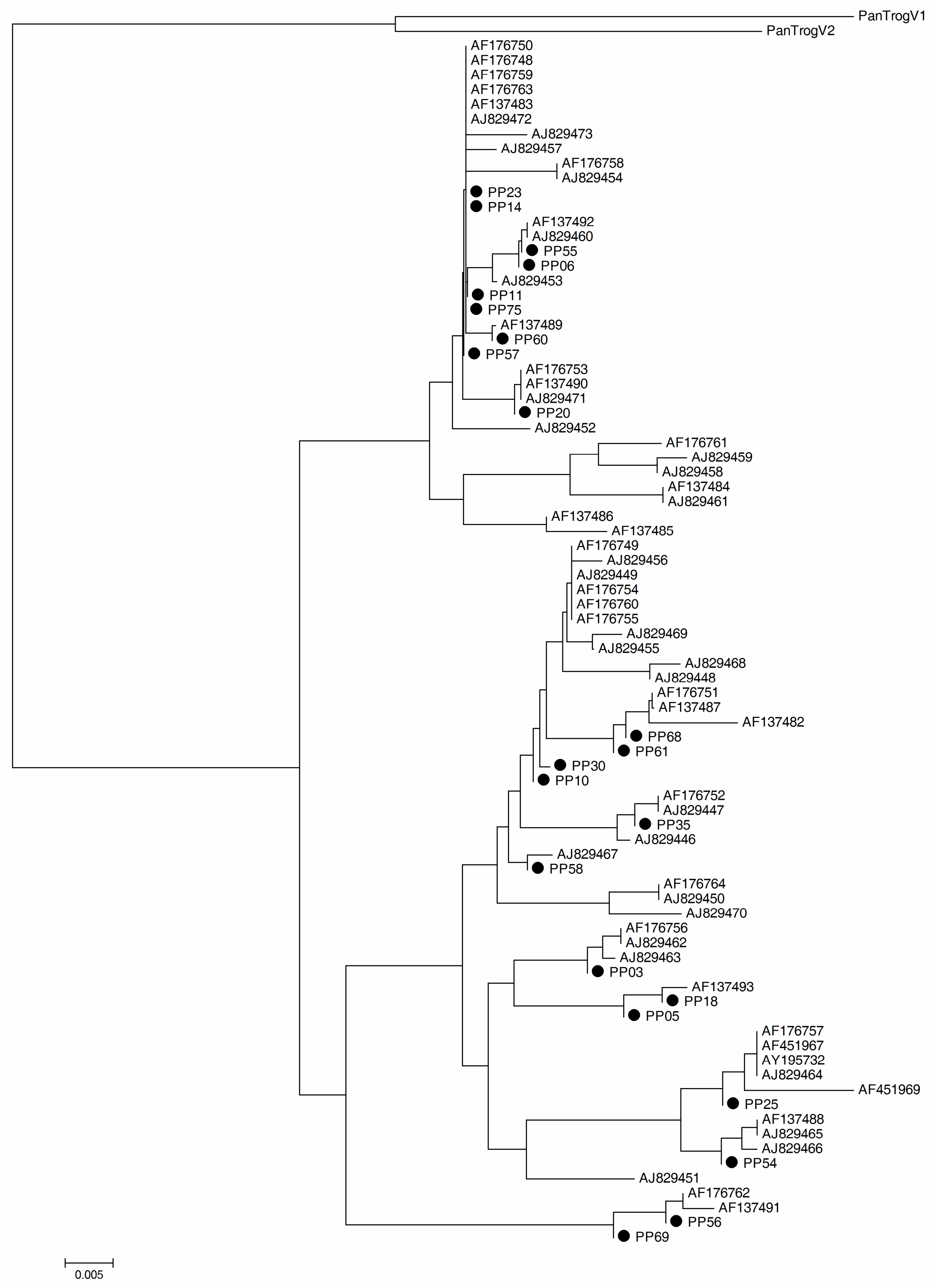

Supplement: Additional file 1 — Figure S1. Neighbor-joining tree of Pan paniscus hypervariable region I sequences using Pan troglodytes as outgroup. Previously published sequences are shown by their GenBank accession numbers. Complete Pan paniscus mtDNA sequences described in this study are marked by dots. Scale bar, evolutionary distance (substitutions per nucleotide position). [file 1471-2148-10-270-S1.PDF]
