# Supplementary material for: Distinct patterns of mitochondrial genome diversity in bonobos (Pan paniscus) and humans
Source: BMC Evol Biol. 2010 Sep 2;10:270. doi: 10.1186/1471-2148-10-270 (PMC2942848; doi:10.1186/1471-2148-10-270)

Additional file 2 Figure S2 - Detailed phylogenetic tree of complete *Pan paniscus* mtDNA sequences displaying all detected polymorphic positions. For each branch, strictly branch-specific mutations are listed on the left-hand side of the line, while homoplasic mutations (occurring also in other independent branches) on the right-hand side. Stars indicate the lack of the specific bonobo-allele (representing a 'backwards' mutation to the human reference). A list of such 'backwards' mutating sites is shown at the root (top). PPRefCOD and PPRefDL, coding and D-loop regions of the only previously reported complete *Pan paniscus* mtDNA sequence [GenBank:NC_001644].

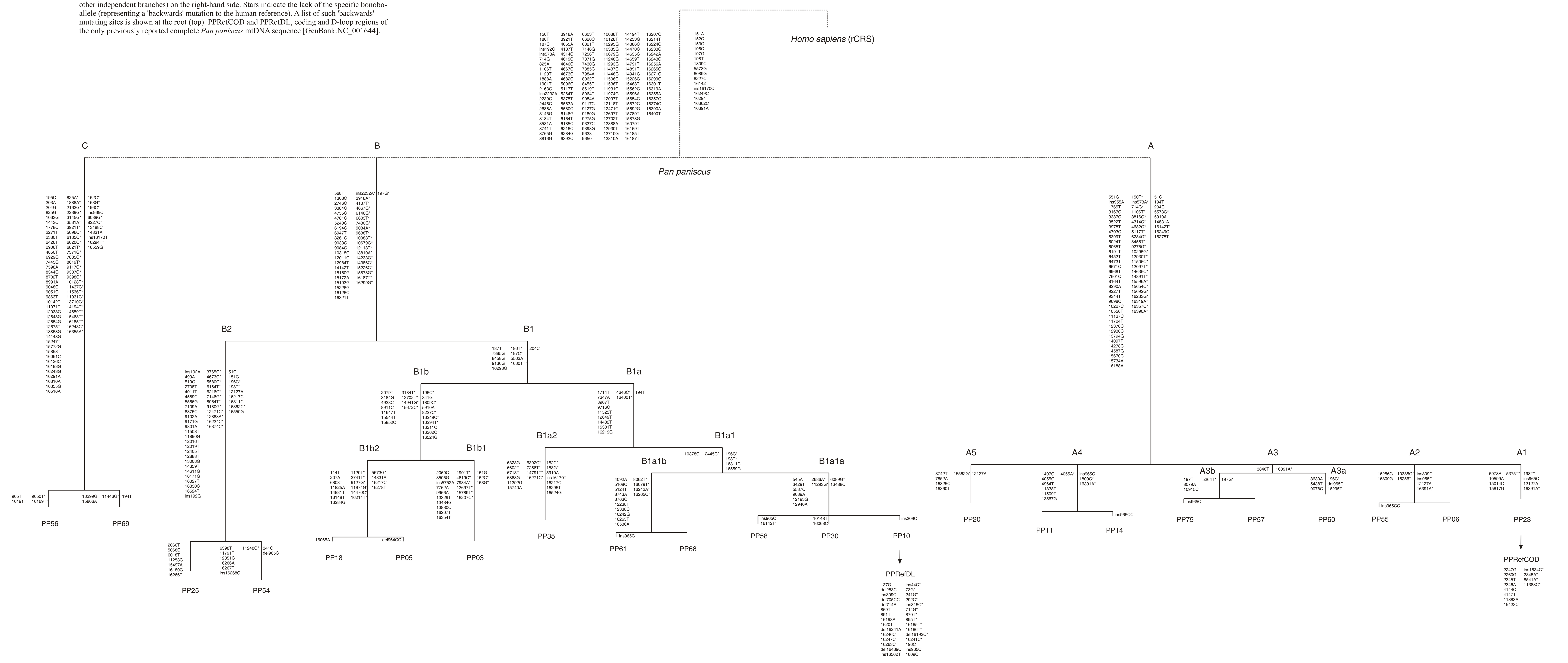

Supplement: Additional file 2 — Figure S2. Detailed phylogenetic tree of complete Pan paniscus mtDNA sequences displaying all detected polymorphic positions. For each branch, strictly branch-specific mutations are listed on the left-hand side of the line, while homoplasic mutations (occurring also in other independent branches) on the right-hand side. Stars indicate the lack of the specific bonobo-allele (representing a 'backwards' mutation to the human reference). A list of such 'backwards' mutating sites is shown at the root (top). PPRefCOD and PPRefDL, coding and D-loop regions of the only previously reported Pan paniscus mtDNA sequence [GenBank:NC_001644]. [file 1471-2148-10-270-S2.PDF]
