# Supplementary material for: Distinct patterns of mitochondrial genome diversity in bonobos (Pan paniscus) and humans
Source: BMC Evol Biol. 2010 Sep 2;10:270. doi: 10.1186/1471-2148-10-270 (PMC2942848; doi:10.1186/1471-2148-10-270)

Additional file 3 Figure S3 - Ratios of non-synonymous to synonymous mutations in mitochondrial protein coding genes. d_N/d_S ratios of within-group polymorphic sites in the mitochondrial encoded subunits of complexes I, III, IV and V in *Pan paniscus* and diverse human haplogroups. Numbers of analyzed individual sequences are shown in brackets.

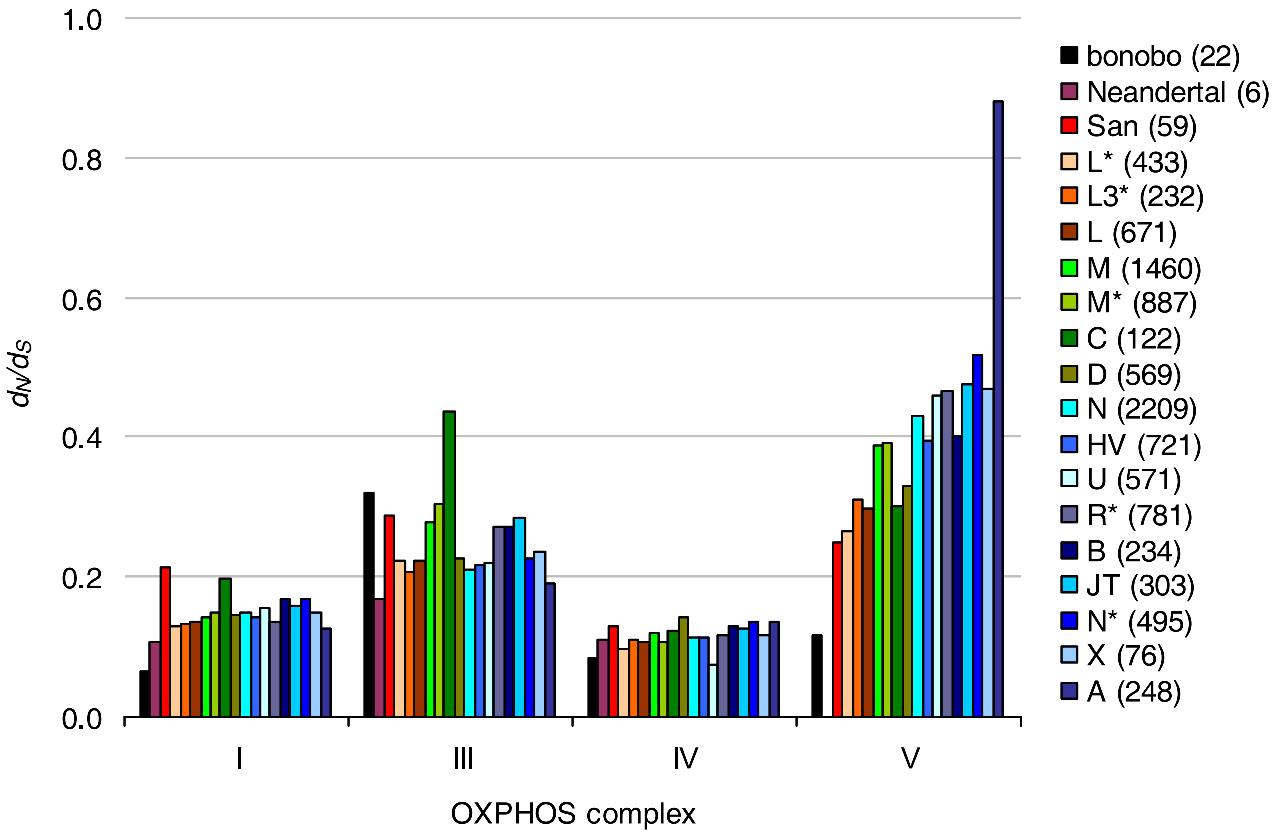

Supplement: Additional file 3 — Figure S3. Ratios of non-synonymous to synonymous mutations in mitochondrial protein coding genes. dN/dS ratios of within-group polymorphic sites in the mitochondrial encoded subunits of complexes I, III, IV and V in Pan paniscus and diverse human haplogroups. Numbers of analyzed individual sequences are shown in brackets. [file 1471-2148-10-270-S3.PDF]
