# Supplementary material for: Distinct patterns of mitochondrial genome diversity in bonobos (Pan paniscus) and humans
Source: BMC Evol Biol. 2010 Sep 2;10:270. doi: 10.1186/1471-2148-10-270 (PMC2942848; doi:10.1186/1471-2148-10-270)
Supplement: Additional file 4 — Table S1. Non-synonymous mutations in mitochondrial genes of complex V. Scoring of amino acid changes according to Betts and Russell [44]. Positive values indicate favored changes, zero neutral changes, negative values disfavored changes in membrane proteins. [file 1471-2148-10-270-S4.PDF]

Additional file 4 Table S1 - Non-synonymous mutations in mitochondrial genes of complex V. Scoring of amino acid changes according to Betts and Russell [44]. Positive values indicate favored changes, zero neutral changes, negative values disfavored changes in membrane proteins.

Mutation	Frequency	Gene	Amino acid	Score
Bonobo (N = 22)				
8702T	2	ATP6	T59I	0
8743A	2	ATP6	V73M	1
8875C	1	ATP6	F117L	1
9102A	1	ATP6	I192M	1
9127G	20	ATP6	I201V	2
9136G	7	ATP6	I204V	2
Human haplogroup L0a' (N = 59)				
8420G	3	ATP8	T19A	1
8420C	1	ATP8	T19P	-1
8459G	6	ATP8	N32D	6
8508G	1	ATP8	N48S	2
8545A	6	ATP6	A7T	1
8566G	1	ATP6	I14V	2
8641C	1	ATP6	N39H	3
8711G	2	ATP6	N62S	2
9037G	3	ATP6	M171V	1
9136G	14	ATP6	I204V	2
9196A	1	ATP6	D224N	6
Human haplogroup A (N = 248)				
8369T	2	ATP8	P2S	-1
8382T	1	ATP8	T6I	0
8409T	1	ATP8	P15L	-1
8459G	1	ATP8	N32D	6
8460G	2	ATP8	N32S	2
8471T	1	ATP8	P36S	-1
8496C	1	ATP8	M44T	0
8520G	1	ATP8	E52G	3
8548C	5	ATP6	S8P	-1
8551C	1	ATP6	F9L	1
8563G	66	ATP6	T13A	1
8567C	2	ATP6	I14T	-1
8572A	1	ATP6	G16S	1
8602C	1	ATP6	F26L	1
8623G	1	ATP6	T33A	1
8648A	1	ATP6	R41Q	6
8701G	1	ATP6	T59A	1
8705C	1	ATP6	M60T	0
8723A	1	ATP6	R66Q	6
8756C	4	ATP6	I77T	0
8764A	1	ATP6	A80T	1
8852T	1	ATP6	W109L	-2
8857A	2	ATP6	G111S	1
8860G	247	ATP6	T112A	1
8870C	1	ATP6	M115T	0
8884G	1	ATP6	K120E	1
8962G	3	ATP6	T146A	1
9007G	1	ATP6	T161A	1
9058G	1	ATP6	T178A	1
9068C	1	ATP6	M181T	0
9095C	1	ATP6	L190P	-1
