# Supplementary material for: Distinct patterns of mitochondrial genome diversity in bonobos (Pan paniscus) and humans
Source: BMC Evol Biol. 2010 Sep 2;10:270. doi: 10.1186/1471-2148-10-270 (PMC2942848; doi:10.1186/1471-2148-10-270)
Supplement: Additional file 5 — Table S2. Human haplogroup definitions used in the study. Haplogroups defined by the presence (+) or the absence (-) of specific mutations as compared to the revised Cambridge Reference Sequence [18]. [file 1471-2148-10-270-S5.PDF]

Additional file 5 Table S2 - Human haplogroup definitions used in the study.
Haplogroups defined by the presence (+) or the absence (–) of specific mutations as compared to the revised Cambridge Reference Sequence [18].

Haplogroup	Defining mutations
L0a' (San)	+4312T, –5603T, +9042T, +9347G, –15136T
L*	+769A, +1018A, –10400T, +10873C, –14783C
L3*	–769A, –1018A, –10400T, +10873C, –14783C
L	–10400T, +10873C, –14783C
M	+10400T, +10873C, +14783C
M*	–4883T, –5178A, +10400T, +10873C, +14783C
C	+3552A, +13263G, +14318C
D	+4883T, +5178A, +10400T, +10873C, +14783C,
N	–769A, –1018A, –10398G, –10400T, –10873C, –14783C
HV	–11719A, –12705T, –14766T
U	+11467G, +12308G, +12372A
R*	–11467G, +11719A, –12308G, –12372A, –12705T
B	+del8271ACCCCCTCT, –11467G, +11719A, –12308G, –12372A, –12705T, +16189C
JT	+11251G, –12308G, –12372A, –12705T, +15452A
N*	–10398G, –10873C, +11719A, +12705T, +16223T
X	+6221C, +6371T, +13966G, +14470C
A	+663G, +1736G
